# SOCS Proteins in Immunity, Inflammatory Diseases, and Immune-Related Cancer

**DOI:** 10.3389/fmed.2021.727987

**Published:** 2021-09-16

**Authors:** Mohamed Luban Sobah, Clifford Liongue, Alister C. Ward

**Affiliations:** ^1^School of Medicine, Deakin University, Geelong, VIC, Australia; ^2^Institue of Mental and Physical Health and Clinical Translation, Deakin University, Geelong, VIC, Australia

**Keywords:** SOCS, cytokine, immunity, inflammation, cancer

## Abstract

Cytokine signaling represents one of the cornerstones of the immune system, mediating the complex responses required to facilitate appropriate immune cell development and function that supports robust immunity. It is crucial that these signals be tightly regulated, with dysregulation underpinning immune defects, including excessive inflammation, as well as contributing to various immune-related malignancies. A specialized family of proteins called suppressors of cytokine signaling (SOCS) participate in negative feedback regulation of cytokine signaling, ensuring it is appropriately restrained. The eight SOCS proteins identified regulate cytokine and other signaling pathways in unique ways. SOCS1–3 and CISH are most closely involved in the regulation of immune-related signaling, influencing processes such polarization of lymphocytes and the activation of myeloid cells by controlling signaling downstream of essential cytokines such as IL-4, IL-6, and IFN-γ. SOCS protein perturbation disrupts these processes resulting in the development of inflammatory and autoimmune conditions as well as malignancies. As a consequence, SOCS proteins are garnering increased interest as a unique avenue to treat these disorders.

## Cytokine Signaling in Immunity and Its Regulation

### Overview

Appropriate immune cell development and function relies on the complex interplay between various cell lineages, much of which is mediated by cytokines. These include the interleukin (IL)-2 family, comprising IL-2, IL-7, IL-9, IL-15, and IL-21, that play important roles in the development of specific lymphoid lineages (1, 2), the IL-3 family, IL-3, IL-5, and granulocyte/macrophage colony-stimulating factor (GM-CSF) (3) and members of the IL-6 family, especially IL-6, IL-10, and granulocyte-CSF (G-CSF) (4), that regulate myeloid lineage development and also impact on inflammation, as well as the interferons (IFNs), that mediate antiviral responses and modulate immune cell development and inflammation. Signaling by cytokines needs to be tightly controlled in order to ensure the immune system is maintained at homeostatic levels, as this could result in the development of inflammatory conditions, increased susceptibility to infectious disease and for pathologies as well as an increased propensity to develop cancer (5–7).

### Cytokine Signaling and Its Control

**Figure 1:**
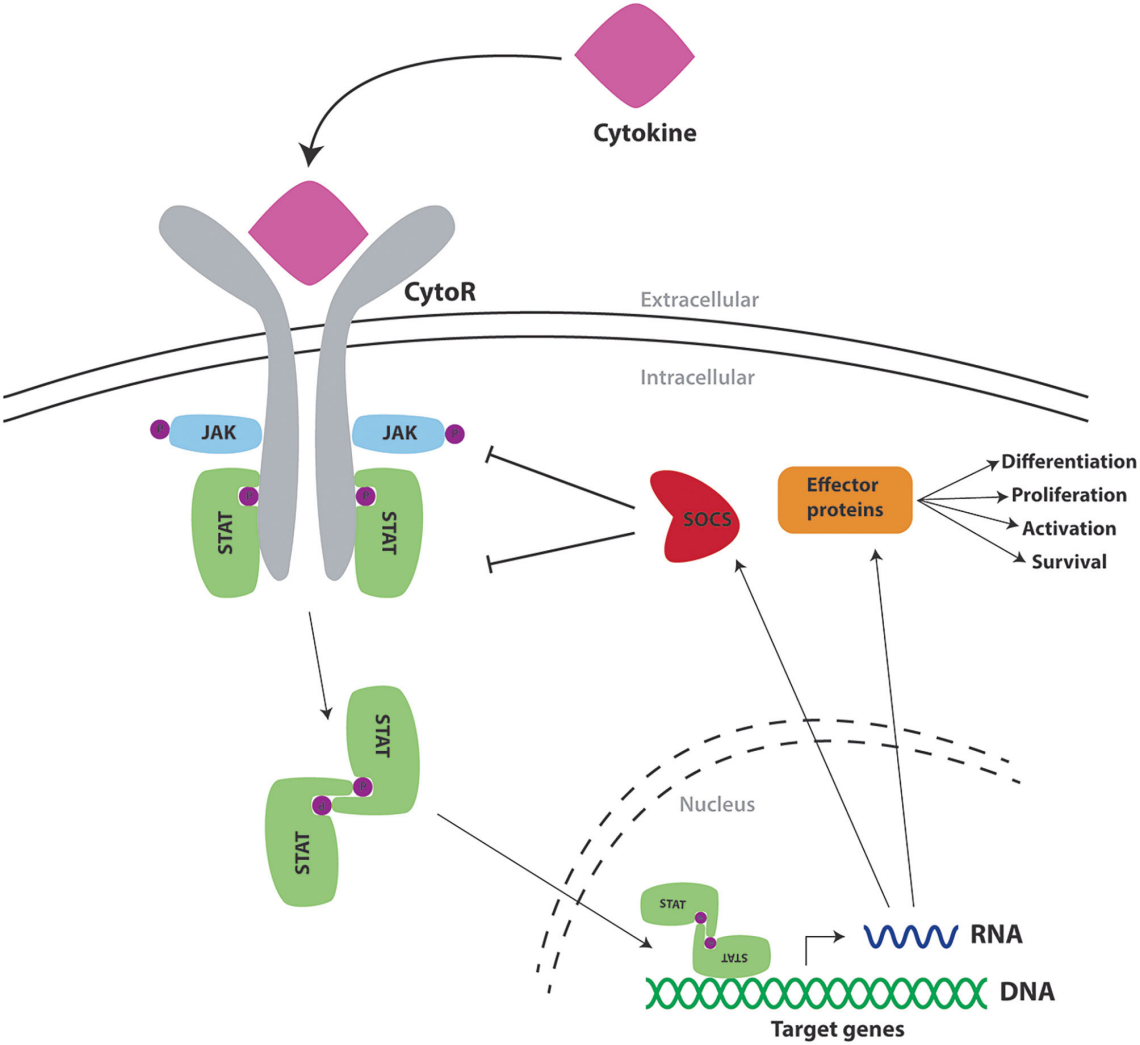
Cytokine signaling via the JAK/STAT pathway and its regulation by SOCS proteins. Schematic representation of a cytokine (pink) binding to the extracellular portion of its cognate cytokine receptor (CytoR—gray), to cause activation of associated intracellular Janus kinases (JAK—blue), which mediate phosphorylation (P—purple) of tyrosine residues found on components of the receptor complex. These form docking sites for signal transducer and activator of transcription proteins (STAT—green), which also undergo phosphorylation to form STAT dimers. These translocate into the nucleus to activate transcription of target genes encoding effector proteins (orange) as well as SOCS proteins (red) that provide negative feedback regulation.

Cytokines bind to cell surface receptors on responsive cells triggering a series of intracellular events that ultimately alter the state of the cell through the stimulation of important genes (8). The majority of cytokine receptors utilize the Janus kinase (JAK)/Signal transducer and activator of transcription (STAT) pathway in order to transmit signals to the nucleus (Figure 1) (6). Mammals possess four JAKs (JAK1, JAK2, JAK3, and TYK2) (9) and seven STATs (STAT1, STAT2, STAT3, STAT4, STAT5A, STAT5B, and STAT6) (10), with specific cytokine receptors using a particular combination of these to influence immune cell production and function. In response to cytokine binding to specific receptors, a phosphorylation cascade is initiated resulting in tyrosine phosphorylation of STATs found in the cytoplasm (11). Upon phosphorylation, STATs dimerize through reciprocal interactions, with STAT dimers subsequently entering the nucleus *via* an active process involving importin proteins (12) and GTPases such as Rac1 and Ran (13, 14), allowing them to initiate transcription of target genes (15).

A variety of proteins are involved in the negative regulation of cytokine receptor signaling through the JAK/STAT pathway such as SH2-containing phosphatases (SHPs), protein inhibitors of activated STATs (PIASs) and suppressors of cytokine signaling (SOCS) proteins (16). Both SHP and PIAS proteins are present in the cytosol prior to the onset of signaling. SHPs regulate signaling through mediating dephosphorylation of various pathway components following recruitment *via* their SH2 domain (17). Alternatively, PIASs inhibit the activity of STATs at various levels and thereby dissipate signaling (17). In contrast, SOCS proteins are negative feedback regulators that are induced directly by STAT proteins and in turn act to negatively regulate the JAK/STAT pathway *via* various mechanisms to ensure appropriate signal dissipation (18).

## SOCS Proteins

**Figure 2:**
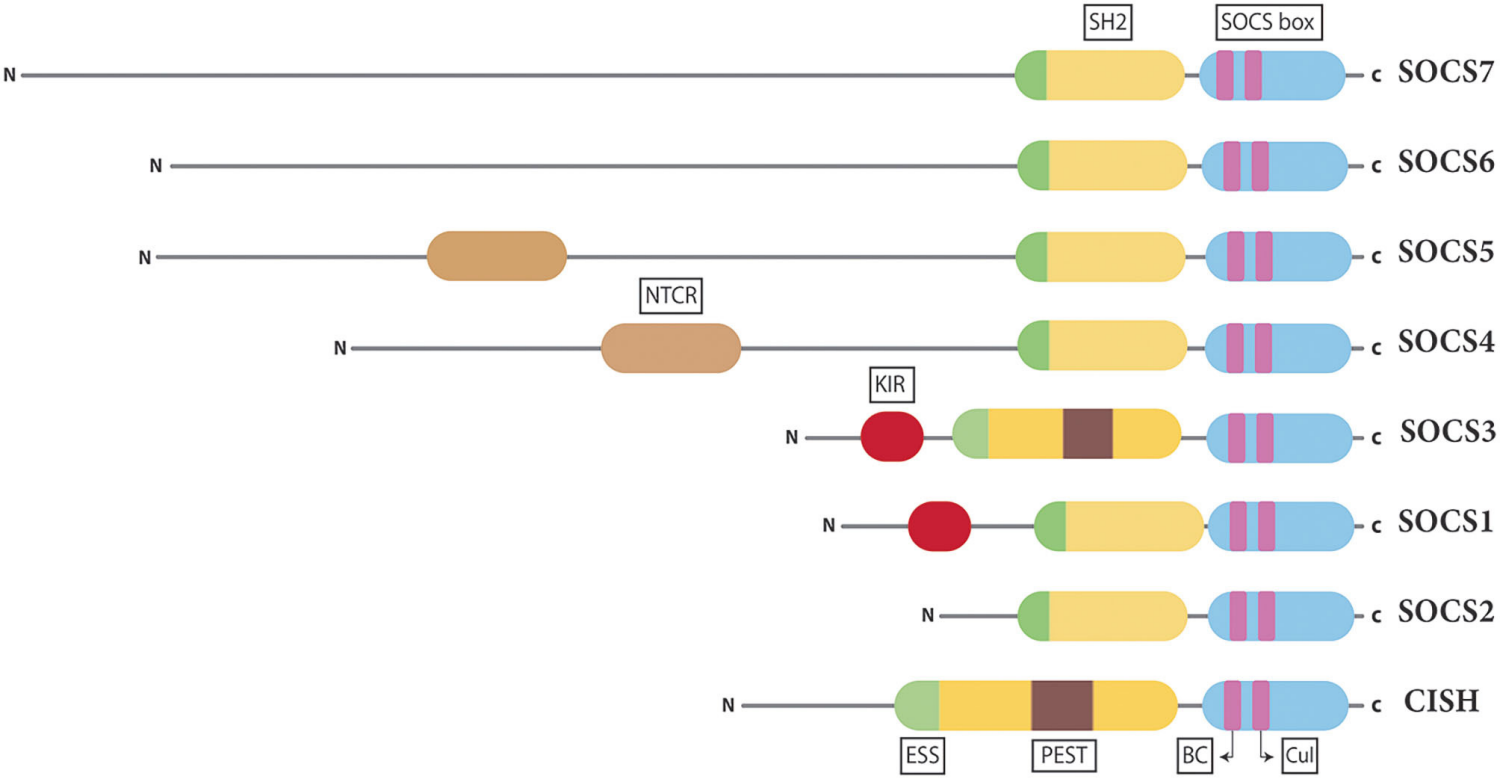
Structure of the SOCS proteins. Schematic representation of the eight mammalian SOCS proteins presented in pairs based on conserved structure and function. Shown are the universal central SH2 domain (yellow), including a unique extra α-helical structure termed the extended SH2 domain (ESS) (green) found in all SOCS proteins and a PEST motif (purple) found in SOCS3 and CISH as well as the C-terminal SOCS box domain (blue), containing Elongin BC (BC) and Cullin (Cul) boxes (pink). In addition, there is a variable N-terminal domain, containing a specialized kinase inhibitory region (KIR) in SOCS1 and SOCS3 (red) and an N-terminal conserved region (NTCR) in SOCS4 and SOCS5 (brown).

There are eight human SOCS proteins, SOCS1–7 and cytokine inducible SH2-containing protein (CISH) (Figure 2) (19). All SOCS proteins show conserved structural similarities and mechanisms of action but with unique aspects for different family members (16, 20), with pairs of SOCS proteins showing greater similarity reflecting their evolutionary history (21). Indeed, there is strong conservation of SOCS proteins, with other mammalian species harboring an equivalent complement and other higher vertebrates having homologs for each, with additional duplicates in teleost fish (22). Invertebrates such as Drosophila also possess multiple SOCS proteins that also participate in the regulation of cytokine and other signaling (23), highlighting the importance of these negative regulators within the cytokine receptor/JAK/STAT pathway.

### Structure of SOCS Proteins

All SOCS family members contain two conserved regions organized in a signature arrangement comprising a central Src-homology 2 (SH2) domain and a C-terminal SOCS box domain (Figure 2) (24). The SH2 domain allows SOCS proteins to bind target substrates through interactions with phosphorylated tyrosine residues (25). However, unlike the canonical SH2 domain found in many signaling proteins, those of SOCS proteins contain an N-terminal α-helical extension, called the extended SH2 domain (ESS) (26). The SOCS box is able to assemble components of an E3 ubiquitin ligase complex *via* two motifs, the Elongin B/C (BC) box and the Cullin (Cul) box (27, 28). The largest variability in SOCS protein structure is observed at the N-terminus (19). The N-terminal domains of SOCS1–3 and CISH are of similar length, but those of SOCS4–7 are considerably longer (29). Specialized motifs within the N-terminal domains have been identified in related SOCS proteins. SOCS1 and SOCS3 contain a unique kinase inhibitory region (KIR) that is able to bind and inhibit JAKs (30), while SOCS3 and CISH contain a PEST motif between the SH2 domain and SOCS box (26, 31), with SOCS4 and SOCS5 having a distinct N-terminal conserved region (NTCR), the exact function of which is yet to be determined (29).

### Mechanisms of SOCS Action

**Figure 3:**
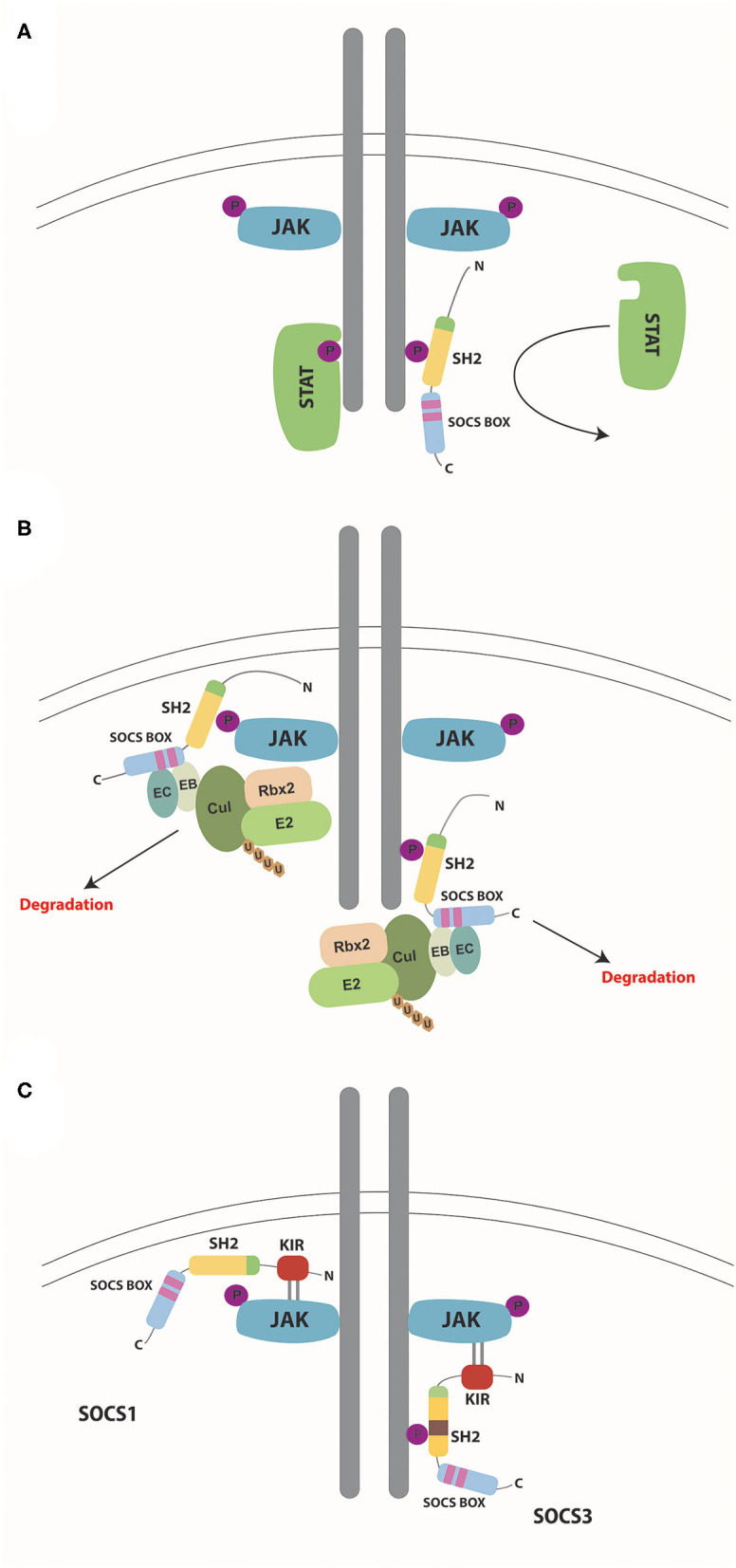
Mechanisms of SOCS action. Schematic representation of the mechanisms by which SOCS proteins negatively regulate cytokine receptor signaling via the JAK/STAT pathway. **(A)** Competitive binding. SOCS proteins bind to phosphorylated tyrosine residues on the cytokine receptor via their SH2 domain, thereby preventing docking of STAT (and other) proteins and so inhibiting their subsequent activation. **(B)** Degradation. SOCS proteins bind through their SH2 domains to phosphorylated target proteins such as receptor-associated JAK proteins (left) or receptors themselves (right), subsequently assembling components of the E3 ubiquitin ligase complex: elongins B and C binding to the BC box and Cullin 5 binding to the Cul box located within the SOCS box. Other components of the complex such as Rbx2 and E2 are assembled through interactions with Cullin 5. Ubiquitin molecules are transferred from the ligase to the target protein substrates, which signals their degradation along with that of associated proteins within the complex. **(C)** Inhibition of kinase activity. Both SOCS1 and SOCS3 utilize their SH2 domain to bind to phosphorylated tyrosine residues on either JAKs (left) or receptors (right), respectively. Both then directly inhibit JAK kinase activity via their KIR domain.

SOCS proteins regulate cytokine receptor signaling through several different mechanisms such as competitive binding, targeting proteins for degradation/re-routing, and inhibition of kinase activity, which utilize different combinations of protein domains and motifs (Table 1). However, in each case the SH2 domain typically allows SOCS proteins to bind to specific components of the cytokine receptor signaling complex or downstream signaling proteins through interactions with appropriate phosphotyrosine containing motifs (Figure 3) (7).

**Table 1:** Mechanisms of SOCS action on cytokine signaling in immune cells.

**SOCS protein**	**Mechanism of action**	**Cytokine**	**STAT**	**References**
SOCS1	JAK inhibition	IFN-γ	STAT1	(32)
		IFN-α/β	STAT1/2	(33)
		IL-12	STAT4	(34)
		IL-2,7,15,21	STAT5	(35–37)
		IL-4	STAT6	(38)
	Competitive binding	IL-2,15	STAT5	(39)
SOCS2	JAK inhibition	IL-2,15	STAT5	(40, 41)
		IL-3, GM-CSF	STAT5	(42)
		IL-4	STAT6	(40)
SOCS3	JAK inhibition	IL-6, 23, G-CSF	STAT3	(43–45)
	Degradation/re-routing	IL-12	STAT4	(46)
		G-CSF	STAT3	(47)
CISH	Competitive binding	IL-12	STAT5	(48)
	Degradation/re-routing	IL-15	STAT5	(49)
		IL-3, GM-CSF	STAT5	(50)
	?	IL-4,13	STAT6	(51)
SOCS5	Competitive binding	IL-4	STAT6	(52)
SOCS4,6,7	?	?	?	

*?, not known*.

### Competitive Binding

SOCS proteins are able to bind to receptor complexes and sterically block binding of other molecules. This is generally through interactions of their SH2 domain to phosphotyrosine motifs to impede binding of other SH2 domain-containing proteins, notably including STATs, thereby inhibiting their activation (Figure 3A), but other mechanisms also exist (7).

### Protein Targeting

SOCS proteins are also able to target proteins, including the receptor and associated JAK and other signaling proteins (24, 53), through formation of an E3 ubiquitin ligase complex (Figure 3B). Elongin BC and Cullin 5 are recruited directly to specific motifs in the SOCS box domain as part of a complex containing Ring box protein 2 (Rbx2) and a ubiquitin conjugating enzyme (E2) (54). Once the E3 ubiquitin ligase complex is assembled, target proteins bound through the SH2 domain undergo ubiquitination, leading to their proteasomal degradation or intracellular re-routing (55).

### Inhibition of JAK Activity

SOCS1 and SOCS3 can also directly inhibit the kinase activity of receptor-associated JAK proteins thereby suppressing signal propagation (Figure 3C) (56). SOCS1 binds to the JAK proteins directly through its SH2 domain (57), whereas SOCS3 instead binds to the associated receptor (57, 58). In each case the respective KIR motif is then able to act as a pseudo-substrate thereby blocking the substrate-binding groove of the JAK kinase domain to inhibit its activity (57).

### Regulation of SOCS Proteins

SOCS protein levels have been shown to be regulated by multiple layers of control. At the transcriptional level, SOCS genes—particularly *SOCS1, SOCS2, SOCS3*, and *CISH*—are strongly induced by activated STATs (59), but other transcription factors can additionally mediate this (60). SOCS genes can also repressed by others, as described for *SOCS1* and *SOCS3* by growth factor independence-1 (GFI-1) through promoter binding (61, 62), and can be silenced epigenetically through regulation through methylation of CpG islands or histone deacetylation (63–65). SOCS mRNAs are typically short-lived, with AU_2−4_A motifs in the 3′UTR linked to stability (31, 66), along with m^6^A methylation (67). Furthermore, the 5′UTR of the SOCS1 mRNA has an inhibitory effect on translation mediated by an upstream open reading frame (68), and can also influence translation of different protein isoforms (69). In addition to this, translation is controlled by a myriad of micro-RNAs (miRNAs) that typically bind to sites located in the 3′UTR (70–74). SOCS proteins are also relatively unstable, with binding to elongins and phosphorylation of the SOCS box found to influence relative protein stability (75–77). CISH and SOCS3 also possess a PEST motif adjacent to their SH2 domain, which provides an additional mechanism to impact protein stability (26, 31).

## SOCS Proteins as Regulators of Immunity and Its Perturbation in Disease

**Figure 4:**
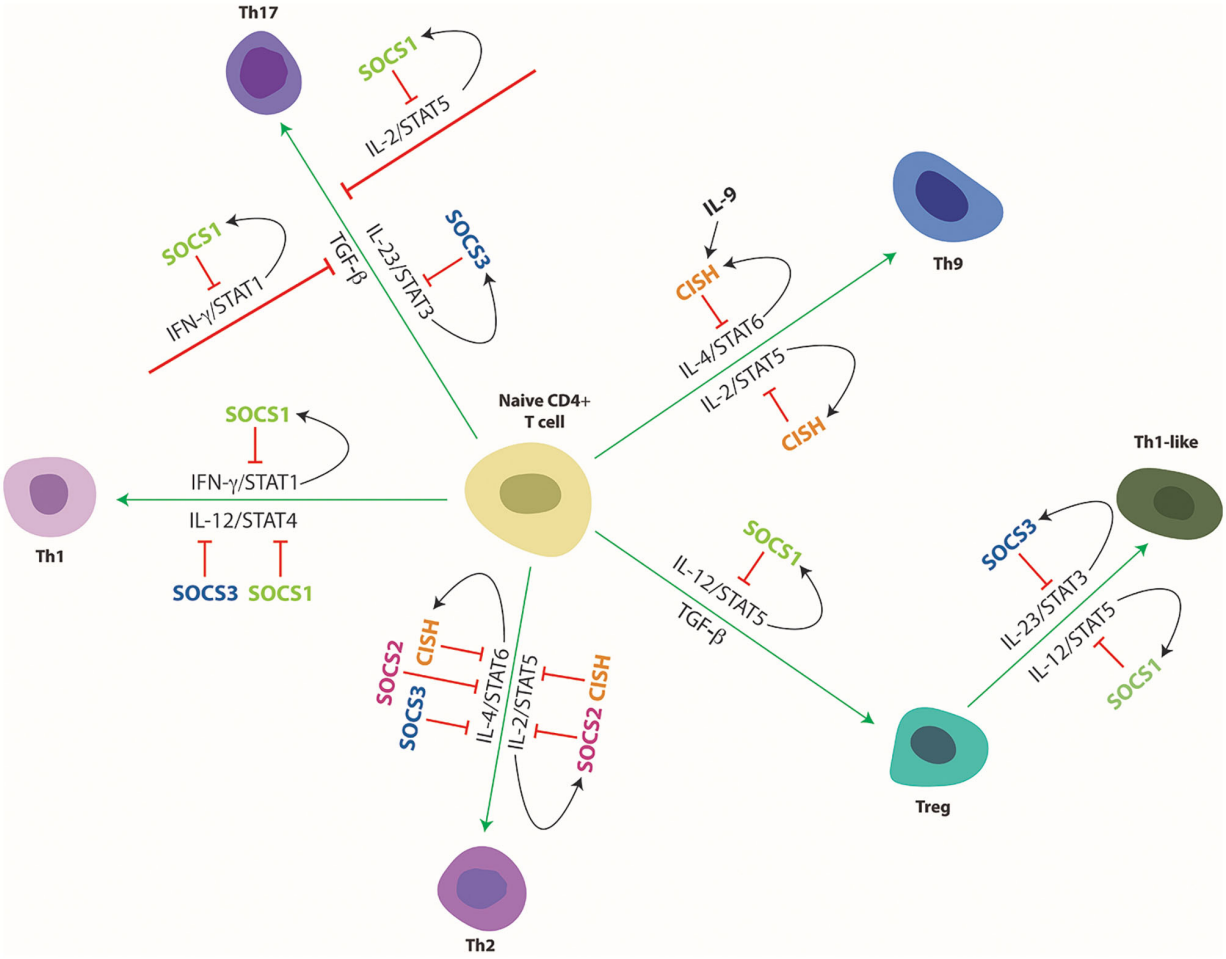
Cytokine mediated differentiation of naïve CD4+ T cells to their various subsets and its regulation by SOCS proteins. Schematic representation of naïve CD4+ T cell differentiation toward the indicated subtypes and its control by specific cytokines and downstream STAT proteins and their regulation by specific SOCS proteins. Green arrows indicate stimulation and red lines indicate suppression of developmental pathways, with black arrows delineating SOCS induction.

**Figure 5:**
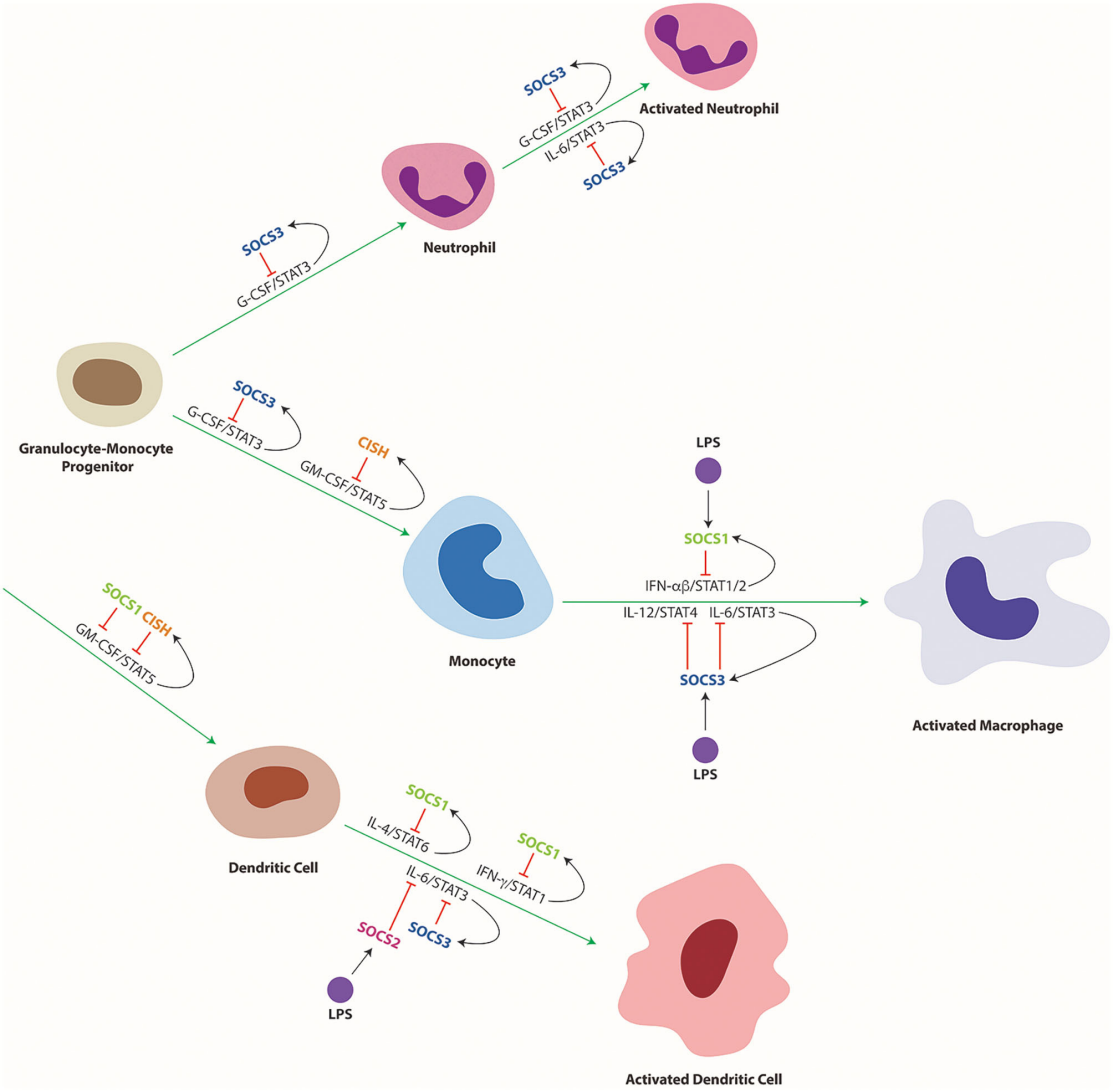
Cytokine mediated differentiation and activation of myeloid cells and its regulation by SOCS proteins. Schematic representation of myeloid cell differentiation along the indicated lineages, including their subsequent activation as well as its control by specific cytokines and downstream STAT proteins and their regulation by specific SOCS proteins. Green arrows indicate stimulation and red lines indicate suppression of developmental pathways, with black arrows delineating SOCS induction.

SOCS proteins, particularly, SOCS1–3 and CISH are closely involved in the regulation of cytokine receptor signaling (19, 78). Since cytokines play a key role in immune cell development and function, SOCS proteins influence multiple aspects of immune system development and function, notably including helper T (Th) cell differentiation (Figure 4) and myeloid cell development and function (Figure 5). They are also implicated in a range of inflammatory and autoimmune diseases as well as immune-related cancers.

### SOCS1

SOCS1 is induced by a wide range of cytokines, including IFN-γ *via* STAT1 and those that utilize the IL-2 receptor common gamma chain (the IL-2Rγ_c_) receptor subunit *via* STAT5 (35). SOCS1 most commonly interacts directly with JAK1, JAK2, and TYK2 including in their dephosphorylated states, with its major mechanism of action being to inhibit the activity of these JAKs through the pseudosubstrate KIR, with the SOCS box of SOCS1 having a poor affinity for E3 ligase components (57). SOCS1 can also bind to phosphotyrosine residues on cytokine receptor chains such as IL-2Rβ through its SH2 domain (39). Through these mechanisms SOCS1 regulates a raft of cytokines central to the control of immunity and inflammation, including IFN-γ and downstream STAT1 (32) and various IL-2R γ_c_ subunit utilizing cytokines and downstream STAT5 and STAT6 (38, 79).

Transgenic expression of SOCS1 in T cells resulted in an increased CD4+/CD8+ T cell ratio and peripheral T cell defects, with reduced IFN-γ-induced STAT1 activation (80). In contrast, SOCS1^−/−^ mice succumbed to early lethality at 2–3 weeks post-gestation due to pathological inflammation characterized by T cell activation (81, 82), which could be substantially alleviated through injection of anti-IFN-γ or by crossing onto a IFN-γ deficient background, suggesting that excessive IFN-γ signaling was largely responsible (32). Transgenic expression of SOCS1 with a deleted SOCS box in the SOCS1^−/−^ mice also facilitated enhanced survival, highlighting the importance of KIR-mediated negative regulation (83). Moreover, the inflammatory phenotype could be mitigated by crossing onto a RAG2 deficient background (81) or in thymocyte-specific SOCS1 knockout mice (84), indicating lymphocytes were responsible for much of the inflammation. However, SOCS1^−/−^ IFN-γ^−/−^ mice still displayed markedly lower numbers of total T cells, reduced CD4/CD8 T cell ratio, and increased T cell activation (85). The T cells in these mice were hypersensitive to a range of cytokines that utilize IL-2Rγ_c_, including IL-2 and IL-4 and IL-7, resulting in enhanced proliferation and survival (84, 85).

SOCS1 influences the development of CD8+ T cells, with both SOCS1^−/−^ and SOCS1^−/−^IFN-γ^−/−^ mice exhibiting a significant increase in CD8+ T cells (86, 87). This was mediated by enhanced signaling by the cytokines IL-7, IL-15, and IL-21 which stimulate CD8+ T cell production (88, 89). In the absence of SOCS1, STAT5 was constitutively activated by IL-7 and IL-15, stimulating pathological generation of CD8+ T cells (79, 86), which was blunted in both SOCS1^−/−^IL-7^−/−^ and SOCS1^−/−^IL-15^−/−^ mice (36). The excessive IL-15 signaling also impaired elimination of auto-reactive CD8+ T cells thereby contributing to autoimmunity (79).

SOCS1 has additionally been shown to control the polarization of CD4+ T cells toward their various subtypes (35) (Figure 4). Induction of SOCS1 by IL-4 and IL-6, as well as by IFN-γ itself, directly inhibits IFN-γ-mediated STAT1 activation that facilitates Th1 differentiation (34, 90). Accordingly, deficiency of SOCS1 in CD4+ T cells resulted in an elevated Th1 response as a direct result of enhanced IFN-γ signaling, but also indirectly through reduced IL-6/STAT3-mediated Th1 suppression mediated by enhanced levels of SOCS3 (87). As a corollary, overexpression of SOCS1 in CD4+ T cells suppressed Th1 differentiation (90). SOCS1 can also inhibit both IL-4-mediated STAT6 activation that drives Th2 polarization, and IL-12-mediated STAT4 that drives Th1 polarization, with loss of SOCS1 resulting in enhanced signaling *via* both pathways and increased Th cell differentiation (91). SOCS1^−/−^ IFN-γ^−/−^ knockout mice were skewed toward a Th2 response, highlighting the role of SOCS1 in the inhibition of Th2 polarization (92). Loss of SOCS1 in CD4+ T cells additionally suppressed Th17 cell development, with enhanced IFN-γ signaling antagonizing transforming growth factor (TGF)-β mediated SMAD activation and also indirectly blunting IL-6 mediated STAT3 signaling (87). SOCS1 has been further demonstrated to suppress STAT5 activation in response to IL-2, providing an alternative mechanism to inhibit differentiation of Th17 cells (93, 94). SOCS1 is also required for the maintenance and activity of Foxp3+ regulatory T (Treg) cells (95). Interestingly several similarities exist between SOCS1^−/−^ mice and the Treg-deficient *scurfy* mice, both of which face premature death 2–3 weeks after birth and display inflammatory phenotypes associated with increased IFN-γ signals (81, 96). SOCS1 is highly expressed in Treg cells, but T cell-specific over expression of SOCS1 was associated with decreased Treg cells whereas T cell-specific knockout resulted in increased Tregs in the thymus (97). This was due at least partially to the ability of SOCS1 to inhibit IL-2-mediated STAT5 activation that contributes to the proliferation of these cells (97, 98). SOCS1^−/−^ mice were however deficient in peripheral Treg cells despite the enhanced thymocyte development, with adoptive transfer of SOCS1^+/+^ Treg cells able to reduce inflammation and increase survival (99). Treg-specific SOCS1 ablation resulted in a loss of suppressor functions (98). SOCS1^−/−^ Treg cells also showed hyperactivation of STAT1 and STAT3, enhancing the production of IFN-γ and IL-17, respectively, with Foxp3 expression rapidly lost, and these cells converting to Th1- or Th17-like cells (95). This decrease in Foxp3 expression was rescued by deletion of IFN-γ, showing that SOCS1 inhibits IFN-γ mediated STAT1 activation that would otherwise suppress Foxp3 expression (95). In addition, SOCS1 is required in Treg cells to suppress the IL-12-mediated activation of STAT4, which modifies the Treg cells into an inflammatory Th1-like cell (34).

SOCS1^−/−^ mice showed altered natural killer T (NKT) cells, with a decrease in invariant NKT cells but an increased activation of conventional NKT cells that supplemented the inflammation and autoimmunity observed in these mice (84). This was due to enhanced IFN-γ activation and IL-2 and IL-15 proliferative signaling (100). NKT cells displayed sustained IFN-γ and IL-4 signaling in the absence of SOCS1, including loss of the cross-inhibitory action of IFN-γ on IL-4. This resulted in enhanced NKT cell activation that stimulated inflammation, which could be alleviated either by removing NKT cells or by deletion of STAT1 or STAT6 which are activated by IFN-γ and IL-4, respectively (38).

SOCS1 also influences the differentiation and function of dendritic cells (DCs) to suppress systemic autoimmunity and maintain their tolerogenic phenotype. Restoration of SOCS1 expression in T and B cells of SOCS1^−/−^ mice resulted in accumulation of DCs in the thymus and spleen, and pertubed activation of these cells due to hyperresponsiveness to IFNγ and IL-4, which resulted in aberrant B cell expansion and autoreactive antibody production (101). Autoimmunity in SOCS1^−/−^ mice was largely mediated by a specific increase in CD8+ DCs, resulting in increased IFNγ and IL-12 production (102). DCs from SOCS1^−/−^ mice exhibited stronger Th1 responses, also including increased IFNγ production by T cells (103). The mechanisms by which SOCS1 regulates DC development remain controversial, but SOCS1 expression can block GM-CSF-mediated signaling and differentiation of DCs, with SOCS1 shown to induce the ubiquitin-mediated degradation of the GM-CSF-R βc subunit (104) (Figure 5).

SOCS1^−/−^ mice additionally display increased numbers of eosinophils and macrophages, although this appears to be largely an indirect effect of aberrant T cells in these mice rather than impacts on their development *per se*. SOCS1 is rapidly induced by Toll-like receptors (TLR) in response to ligands such as lipopolysaccharide (LPS) and has been found to influence the activation and subsequent polarization of macrophages (105). Indeed, SOCS1^−/−^ mice challenged with LPS show augmented innate immune responses characterized by enhanced macrophage activation, including high levels of tumor necrosis factor (TNF)-α and IL-12 (106, 107). However, the mechanism of action remains somewhat controversial. Several studies have indicated that the effect is indirect, with the SOCS1 induced by TLRs blocking downstream IFNα/β signaling (33, 108). In contrast, others have shown that SOCS1 can directly negatively regulate TLR signaling through interactions with various adapter proteins that such as IL-1 receptor associated kinase (106) and Mal/TIRAP (109) which are responsible for the induction of inflammatory genes *via* NF-κB. Intriguingly, even in the absence of IFN-γ and STAT1, SOCS1^−/−^ mice were hypersensitive to LPS, showing that this regulation is at least partially distinct from the effect of SOCS1 on IFN signaling (106). Either way SOCS1 plays significant immunosuppressive roles in the myeloid lineage mitigating the development of excessive inflammation (110).

With the strong role of SOCS1 in the regulation of multiple aspects of immunity, it is perhaps not surprising that SOCS1 impacts on a variety of inflammatory diseases. T- and NK-cell specific SOCS1 knockout mice displayed increased sensitivity to ConA-induced hepatitis, due hyperresponsive of these cells to IL-2 and IL-15 (100). SOCS1^+/−^ mice and those with T cell-specific SOCS1 ablation were more susceptible to experimentally-induced colitis due to enhanced IFNγ/STAT1-mediated suppression of Treg cells (111). SOCS1^−/−^ mice also showed enhanced formation of atherosclerotic plaques due to increased M1 macrophages and neutrophils (112). In contrast, T cell-specific SOCS1 knockout mice were resistant to experimental autoimmune encephalomyelitis (EAE) in this case a result of enhanced IFNγ/STAT1-mediated suppression of Th17 cells that drive this model of autoimmunity (87). Moreover, transgenic SOCS1 expression in T cells resulted in the development of intestinal inflammation (113). Mice with DC-specific SOCS1 ablation, showed a skewed immune response to bacterial antigen, with reduced CD8+ T cells and NK cells but enhanced innate responses (114). Knockdown of SOCS1 in DCs resulted in reduced susceptibility to *Candida albicans*, with increased phagocytosis and killing by DCs (115), but also increased Th1 cells and elevated serum IFN-γ that blunted inflammation during late stages of infection (116). Altered SOCS1 expression has been observed in patients with systemic lupus erythematosus (117) and rheumatoid arthritis (118), with SOCS1 polymorphisms demonstrated to predict severity of the latter disease (119). Recently, heterozygous loss-of-function germline mutations in SOCS1 have been shown to be associated with early onset human autoimmune diseases, with lymphocytes showing hyperactivation concomitant with increased STAT activation in response to IFN-γ, IL-2, and IL-4 that is reverted with a JAK1/2 inhibitor (120).

SOCS1 has additionally been implicated in a variety of immune cell malignancies, with silencing of SOCS1 due to methylation or mutation commonly reported, reflective of the negative regulatory roles played by SOCS1 in the cytokine-mediated proliferation, differentiation and survival of immune cells (121, 122). For example, SOCS1 is frequently methylated in cases of acute (AML) and chronic myeloid leukemia (CML) (123–125), which is thought to block the ability of SOCS1 to negatively regulate JAK2 activity through kinase inhibition (126), thereby promoting activation of STAT3 and STAT5 which are major drivers of leukemia development (123). However, SOCS1 deficiency can also impact on therapeutic responses, being associated with poor outcomes to IFN-α treatment in AML (127). In CML, SOCS1 is strongly induced by BCR-ABL, but its role is complex, since it can inhibit both pro-proliferative responses mediated by IL-3 and IL-6, as well as anti-proliferative responses to IFNs. In a mouse model, BCR-ABL-dependent growth was resistant to SOCS1 expression, but a subset of SOCS1-expressing mice showed extended disease latency or indeed an absence of disease (128). SOCS1 methylation has also been observed in multiple myeloma, where it correlated with enhanced STAT3 activation and was associated with poorer prognosis (129). Loss-of-function somatic mutations of SOCS1 have also been frequently observed in a range of B cell malignancies. These include Hodgkin's lymphoma (130–132), where they are associated with shorter patient survival (133), SOCS1 deficiency in this disease correlated with hyperactivation of JAK2 and downstream STAT6 leading to excessive proliferation (134), with SOCS1 mutations synergizing with presumed gain-of-function STAT6 mutations (135). Somatic SOCS1 mutations have also been commonly found in diffuse large B cell lymphoma (DLCBL) (131, 136), where they contribute to unrestrained IL-6 signaling (137). Interestingly, patients with such mutations responded less well to anti-CD20 therapy resulting in reduced survival (138). Similar loss-of-function mutations have been reported in gray zone lymphoma (139), primary mediastinal B cell lymphoma (PMBCL) (140) and HIV-associated plasmablastic lymphoma (141). SOCS1 has also been found to be mutated in malignancies involving other lymphoid malignancies, including T cell prolymphocytic leukemia (T-PLL) (142), NK/T cell lymphoma (143), enteropathy-associated T cell lymphoma (EATL) (144), as well as mycosis fungoides, a cutaneous T cell lymphoma (CTCL) (145). Indeed, in CTCL cells, SOCS1 knockdown was shown to increase aggressiveness, with cooperation seen with activating JAK3 mutations (93).

### SOCS2

SOCS2 is strongly activated by and in turn negatively regulates STAT5 and to a lesser extent STAT6 and STAT3 (146, 147). The negative regulation is principally mediated by SOCS box-dependent degradation of target substrates (148), including receptors and downstream signaling molecules (149). Intriguingly SOCS2 also regulates other SOCS proteins, particularly SOCS1 and SOCS3 (150, 151), but in a dose-dependent manner (152). Through this mechanism SOCS2 can actually indirectly stimulate cytokine-induced STAT activation by removing the negative regulation mediated by those SOCS proteins targeted (151). SOCS2 is primarily involved in regulation of developmental and homeostatic pathways, such as those mediated by growth hormone, insulin-like growth factor and prolactin signaling (153, 154). However, more recent studies have illuminated novel functions of SOCS2 within the immune system.

SOCS2^−/−^ mice showed no obvious basal defects in T cells (40, 155–157). However, SOCS2 has been demonstrated to influence lymphoid cell activation and polarization (Figure 4). In the absence of SOCS2, TCR stimulation of CD4+ T cells resulted in enhanced levels of IL-4, IL-5 and IL-13 thereby stimulating the differentiation of Th2 cells at the expense of Th17 cells (40). SOCS2 was also found to be induced by IL-4 and IL-6, with loss of SOCS2 leading to enhanced activation of STAT5 and STAT6 in response to IL-2 and IL-4, respectively, but blunted IL-6-mediated STAT3 activation potentially mediated indirectly by SOCS1 and SOCS3 which were upregulated in SOCS2^−/−^ mice (40). Thymic Foxp3^+^ Treg cells were unaffected in SOCS2^−/−^ mice, but enhanced IL-4-induced STAT6 peripheral inducible Treg cells (iTreg) were observed, resulting in increased levels of inflammatory cytokines such as IL-13 and IFN-γ (156). This suggests that SOCS2 is required to conserve the intrinsic anti-inflammatory phenotype of iTreg cells (156).

NK cells were elevated in the bone marrow and spleen of SOCS2^−/−^ mice (41). This was shown to be a cell autonomous effect, with SOCS2 able to inhibit IL-15 signaling specifically through the JAK2/STAT5 pathway, but not the JAK1/STAT3 pathway, mediated *via* direct interaction with JAK2. This caused increased NK cell differentiation and although these cells showed unaltered cytolytic activity and IFNγ secretion, SOCS2^−/−^ mice showed enhanced resistance to melanoma (41). SOCS2 was also found to be induced by IL-15 in human NK cells, although in this case it was found to target phosphorylated proline-rich tyrosine kinase 2 for proteasomal degradation, leading to enhanced cytolytic activity and other effector functions, including secretion of inflammatory cytokines, in these cells (158).

SOCS2 deficiency also impacted on hematopoietic stem cells (HSCs) (42). In these cells, SOCS2 was induced by IL-3, GM-CSF, and thrombopoietin (TPO) *via* STAT5 activation in HSCs, with proliferation mediated by these cytokines increased in HSC derived from SOCS2^−/−^ mice. There was also unrestrained myelopoiesis following bone marrow ablation in these mice, which leads to exhaustion of long-term HSC (42).

Expression of SOCS2 has been shown to be markedly increased during maturation of DCs, both mouse (159) and human (160). Even though its role in DC production is unclear, SOCS2 is induced by ligands such as LPS and Lipoxin A_4_ that signal through TLR4 and TLR2, respectively (161, 162), although this is probably caused by indirect induction of SOCS2 stimulated by type 1 IFNs produced in DCs in response to LPS in a unique autocrine-paracrine loop (161). Indeed, SOCS2 is required for the propagation of MyD88-dependent and -independent signaling pathways downstream of TLR4 (160). As a result, in the absence of SOCS2, LPS induced expression of inflammatory cytokines such as TNFα, IL1-β, and IL-6 was markedly diminished (160). However, another study showed SOCS2 suppression in DCs caused hyperphosphorylation of STAT3 resulting in an increase in inflammatory cytokines (163). In contrast, SOCS2 did not influence TLR signaling in mouse macrophages (33).

SOCS2 has been shown to suppress inflammation in a range of disease models, but the cellular mechanism differs substantially depending on the model. Thus, in models of atopic dermatitis and allergen-induced airway inflammation SOCS2^−/−^ mice showed enhanced allergic responses with increased IgE, eosinophilia and inflammatory pathology attributable to increased Th2 responses, which were also seen following helminth challenge (40). However, in EAE, SOCS2 deficiency resulted in reduced acute phase damage due to increased Th17 and decreased Th1 and Th2 cells, but exacerbated inflammation during the late phase of disease, characterized by increased Th1 cells and decreased Th2 and Treg cells (164). In a mouse model of cerebral malaria SOCS2^−/−^ mice initially showed reduced parasitemia, however at late stages they showed increased parasitemia associated with increased Th1 and Th17 cells and pro-inflammatory cytokines such as IL-6 and IL-17 with decreased Treg cell activation (165). Interestingly, the aberration in T cell ratios and abnormal cytokine production were alleviated when mice were treated with a nitric oxide synthase (NOS) inhibitor, suggesting a role for SOCS2 in regulating NO synthesis (165). In comparison, SOCS2^−/−^ mice showed reduced parasitemia and decreased IFNγ and TNFα following *T. cruzi* infection, which was associated with increased generation of Treg cells but reduced memory T (Tm) cells (166).

SOCS2 has also been implicated in various malignancies (167, 168), notably including myeloproliferative disorders and leukemias although this can be both pro- and anti- tumorigenic. As a major STAT5 target gene, its expression is strongly increased in a range of myeloproliferative disorders where STAT5 is activated, including BCR/ABL-induced CML (169) and JAK2 V617F-induced disease (170). However, SOCS2 has been shown to be dispensable for the induction and propagation of BCR/ABL-mediated disease (155). In contrast, it was demonstrated to be a negative regulator of JAK2 V617F and was epigenetically downregulated in MPD patients (170). Moreover, SOCS2 expression predicted poor outcomes in pediatric AML (171), while high levels of SOCS2 correlated with progression for both myeloid and lymphoid leukemias (42). In this case SOCS2 expression was under the control of the myocyte-specific enhancer factor 2C and was involved in maintaining the stemness of the leukemias (42). SOCS2 has also been identified as a prognostic signature for the development and progression of AML (172). SOCS2 knockdown reduced growth of AML cells *in vitro* and delayed disease upon transplantation into mouse models, with these cells showing mature myeloid cell markers (172), suggesting a potential oncogenic role for SOCS2.

### SOCS3

SOCS3 is principally induced by cytokines that activate STAT3 *via* JAK2, such as those that utilize the GP130 subunit [for example, IL-6, IL-10, and leukemia inhibitory factor (LIF)], or related receptor chains (G-CSF and leptin) but also by those that activate STAT1 (such as IFN-γ), and STAT5 [such as erythropoietin (EPO)] (173). The major mechanism of action for SOCS3 is through docking to cytokine receptors *via* its SH2 domain allowing the KIR to inhibit the activity of adjacent JAKs, but it can also compete with STAT docking and mediate proteasomal degradation (174, 175). It plays a particularly important role in immunity and inflammation *via* its negative regulation of members of the IL-6 family of cytokines that act *via* STAT3, notably including IL-6 and G-CSF (58).

SOCS3^−/−^ mice display embryonic lethality (176, 177), principally as a result of placental defects caused by excessive LIF signaling, with no direct impact on early hematopoiesis (177, 178). Hematopoietic-specific SOCS3 ablation was able to overcome these effects but resulted in the development of a lethal inflammatory disease, characterized by splenomegaly, pericarditis, hepatitis, and neutrophilia (43). This was largely a macrophage and neutrophil-driven process, although non-hematopoietic tissues have also been implicated (179). Hematopoietic-specific SOCS3 knockout mice display elevated IL-6 (43), and are highly susceptible to IL-1β-mediated inflammation leading to rapid mortality (180). IL-6 deficiency ameliorated the inflammatory disease, including that mediated by IL-1β (179). SOCS3 ablation prolonged STAT3 activation by IL-6 (44, 181), but interestingly also resulted in extended STAT1 activation by IL-6, which promoted a IFN-like response, but IL-10 responses were not surprisingly impacted (181). SOCS3 has been shown to dock directly to the GP130 subunit of the IL-6R to suppress signaling (182), with mice harboring a specific mutation at this site (Y759F) displaying similar splenomegaly, lymphadenopathy, B and T cell defects and autoimmune arthritis to SOCS3-deficient animals (183), highlighting the key role of perturbed IL-6R signaling.

SOCS3 knockout did not affect the overall numbers of CD4+ or CD8+ T cells (45, 184), but SOCS3 is believed to influence the polarization of T helper cells (Figure 4). SOCS3 is able to directly block IL-12-mediated STAT4 activation through competitive binding to STAT4 docking sites on the IL-12Rβ2 subunit (46), with high levels of SOCS3 correlating with reduced activation of STAT4 by IL-12 that induces the production of IFN-γ thereby inhibiting Th1 differentiation and favoring Th2 differentiation (184, 185). Enforced expression of SOCS3 impacted T cell development in the thymus and peripheral T cell homeostasis through impaired IL-7-mediated signaling (186), and also inhibited Th cell proliferation (187), presumably through blocking IL-2 production (188, 189). Despite this, T cell specific deletion of SOCS3 was found to suppress production of both Th1 and Th2 cells, as a result of enhanced IL-10 and TGF-β mediated STAT3 activation, producing an anti-inflammatory response resulting in an increase in regulatory Th3 and Th17 cells (190). The increased Th17 cells appears to be due to the loss of negative regulation of IL-23 signaling by either a feedback loop (45) or by crosstalk from inflammatory cytokines such as IFN-γ and LIF (191, 192), as well as ablation of TGF-β-mediated inhibition of IL-6 signaling (193), which each serve to increase STAT3 activation and thus drive Th17 differentiation. Increased expression of pro-inflammatory genes by macrophages from SOCS3-deficient mice further supports Th1 and Th17 differentiation (194). Deletion of SOCS3 in CD4+ T cells revealed an essential role in maintaining the expression of costimulatory molecules CD28 and CTLA-4 and for the induction of IL-2 upon TCR stimulation, that resulted in expansion of Treg cells at the expense of Th17 and Th1 (184). This is consistent with impaired Treg production and immune suppressive capabilities leading to defective self-tolerance following overexpression of SOCS3 in Foxp3+ Treg cells (195) as well as in SOCS3 transgenic mice (186). Indeed, SOCS3 was also shown to serve as a negative regulator of Foxp3 expression to directly inhibit Treg function (196). SOCS3 is also expressed in NKT cells, in response to cytotoxic cytokines such as IL-10 (197), playing an immunosuppressive role through regulation of cytokine signaling (198). Finally, SOCS3 expression in thymic stromal cells has also been demonstrated to be important for normal T cell development (199).

SOCS3 also plays a role in DCs to regulate their tolerogenic capabilities. IL-6 was shown to induce the expression of SOCS3 in DCs, with SOCS3-deficient DCs exhibiting enhanced STAT3 activation and increased tolerogenic activity compared to their wildtype counterparts (200). In human DCs, SOCS3 was shown to induce proteasomal degradation of the key enzyme indoleamine 2,3-dioxygenase (201), providing an additional mechanism by which it can suppress tolerogenic activities.

SOCS3 has several important functions within the myeloid lineage, controlling the differentiation and activation of both neutrophils and macrophages (Figure 5) (202). In neutrophils, G-CSF induces SOCS3 *via* STAT3 activation (203), facilitating negative feedback regulation to maintain G-CSF signaling at homeostatic levels (204). SOCS3 binds directly to the activated G-CSF receptor (G-CSFR) and inhibits signaling through either suppression of JAK2 activity (58) or by ubiquitination of the G-CSFR thereby stimulating the rerouting of the receptor through lysosomes (47). As a consequence, SOCS3^−/−^ mice exhibit excessive G-CSF signaling resulting in neutrophilia (178). HSC-specific SOCS3 ablation resulted in increased colony size in response to G-CSF, whereas neutrophil-specific ablation resulted in neutrophilia, with enhanced and extended G-CSF signaling and increased survival (205). Disruption of SOCS3 in hematopoietic cells also triggered the development of neutrophilia and inflammatory pathologies in later adulthood, including infiltration of neutrophils into multiple tissues such as liver, lung, and muscle tissue accompanied by elevated IL-6 and G-CSF levels. Stimulation of these mice by G-CSF triggered increased progenitor proliferation and enhanced neutrophilia and increased formation of both macrophage and neutrophil colony formation (43). This ultimately resulted in cerebral infiltration by neutrophils hyperactivated by G-CSF (206). Surprisingly, G-CSF increased the formation of both neutrophil and macrophage colonies (43), with the differentiation of progenitors skewed toward macrophages in response to G-CSF or IL-6 (207).

Within the macrophage lineage there is evidence that SOCS3 has both pro-inflammatory as well as anti-inflammatory roles. SOCS3 suppresses the inflammatory activities of macrophages by providing negative feedback regulation of inflammatory cytokines such as IL-6, IL-1β, and IL-12, with myeloid-specific SOCS3 knockouts showing enhanced and prolonged JAK/STAT signaling, increasing the levels of M1 pro-inflammatory genes (194). Furthermore, LPS and TNF-α are also able to induce SOCS3 to provide additional negative regulation of IL-6-mediated STAT3 activation (208, 209), with bacterial infections associated with enhanced SOCS3 expression in macrophages, presumably as an alternative mechanism to suppress their inflammatory capabilities (210, 211). Hence, it can be predicted that SOCS3 attenuates STAT3 activation thereby promoting macrophage activation *via* positive regulation by TGF-β and IL-6 (212). Independent of this pathway, knockdown of SOCS3 in myeloid cells resulted in an increase in phosphatidyl insotiol-3-kinase (PI3K)-AKT signaling which is known to suppress the production of IL-6 and induction of NF-κB, providing an alternative mechanism for SOCS3 to promote inflammation (212, 213). However, LPS-induced SOCS3 in macrophages also indirectly stimulated the induction of inflammatory genes such as NF-κB, although the mechanism that has not been identified (213, 214). These opposing effects could be attributed to the kinetics of STAT3 signaling, and the antagonistic roles of IL-6 on LPS signaling (211). Lastly, in the absence of SOCS3 the phagocytic abilities of macrophages were found to be heightened, attributed to enhanced IL-6 and IL-12 signaling (215, 216). Intriguingly, PI3K-AKT was found to contribute to the heightened ability of macrophages to undergo phagocytosis by promoting the cytoskeletal rearrangement required for this process, with inhibition of PI3K signaling in SOCS3 silenced cells found to suppress the heighted phagocytic capabilities (217).

A variety of disease models have highlighted critical roles for SOCS3 in both the development and resolution phases of inflammation (218). In mouse models of inflammatory arthritis, SOCS3 deficiency in the hematopoietic compartment caused more severe joint inflammation associated with elevated levels of IL-6 and G-CSF, increased neutrophil numbers and constitutively activated CD4+ T cells and macrophages (180). Mice with myeloid-specific SOCS3 ablation also develop more severe EAE (216, 219) and show enhanced LPS induced sepsis (194) and acute lung injury (220). In addition, they are susceptible to experimentally-induced aortic dissection associated with enhanced STAT3 activation in macrophages and increased inflammation (215). In contrast, CD4+ T cell-specific SOCS3 ablation provided protection from uveitis due to an expansion of Treg cells at the expense of Th17 cells (184). Moreover, acute nephritis was associated with increased SOCS3 activation that facilitated activation of macrophages toward a pro-inflammatory M1 phenotype with increased iNOS production (213). SOCS3 deficiency in either the myeloid or lymphoid lineage independently increased susceptibility to *M. tuberculosis* (202). Ablation of SOCS3 within myeloid cells resulted in enhanced IL-6 signaling which inhibited the secretion of TNF-α and IL-12 required to mediate the development of a robust CD4+ T cell response against the infection (221), as well as increased production of NOS2 and Arg1, leading to higher bacterial loads and exacerbated pulmonary inflammation (210). DC-specific SOCS3 ablation resulted in increased susceptibility to infection as a result of reduced cross-talk with T cells (218). Separately, SOCS3 deficiency in lymphoid cells resulted in an increase in IL-17 secretion during *M. tuberculosis* infection, which mediated enhanced neutrophilic inflammation (221). Hematopoietic-specific SOCS3 deficiency also caused increased sensitivity to lymphocytic choriomeningitis virus-mediated lethality (179). Finally, in mice models of inflammatory arthritis, SOCS3 induction alleviated bone degradation and significantly suppressed the autoimmune inflammation within the joints (222).

As a corollary, SOCS3 has also been implicated in specific human inflammatory diseases. These include inflammatory bowel disease (IBD) and its more severe phenotype, Crohns disease (223, 224), with SOCS3 responsible for suppressing the activation of inflammatory genes induced by STAT3 during IBD (224). In mice, activation of STAT3 and expression of SOCS3 is characteristic for the development of IBD and colitis, with SOCS3 acting as a regulatory mechanism to limit pathogenesis, which is primarily driven through macrophages (225). In addition, inflamed tissue obtained from IBD patients showed an increase in classically activated inflammatory macrophages, with a large majority of these cells displaying an upregulation of SOCS3, a reflection of the roles played by SOCS3 in macrophage polarization (212, 213). Indeed, increased SOCS3 expression predicts mucosal relapse in ulcerative colitis (226). Similarly, SOCS3 expression in human arthritic chondrocytes contributed to cartilage damage during arthritis (227, 228).

Due to its role in controlling the activation of the STAT3 oncogene, SOCS3 is typically considered a tumor suppressor protein, although this is not always the case in immune cancers (229). Loss of SOCS3 expression, particularly *via* promoter methylation, has been identified in a variety of hematopoietic malignancies. Thus, SOCS3 methylation occured at high frequency in BCR-ABL-negative chronic myeloproliferative disease (CMPD) and post-CMPD acute myeloid leukemia (230), as well as chronic lymphoproliferative disease of NK cells (CLPD-NK) (231). SOCS3 hypermethylation in multiple myeloma was associated with drastically shortened patient survival, believed to be due to enhanced responsiveness to IL-6, a major driver of this disease (232). SOCS3 expression was also ablated in Lck LSTRA leukemia, with enforced ectopic expression of SOCS3 reducing cell proliferation and increasing apoptosis in Lck-transformed cells (233). Decreased SOCS3 expression due to methylation was detected in a considerable proportion of mantle cell lymphoma (MCL) patients, with a trend for worse outcomes in this cohort. Enforced re-expression of SOCS3 reduced IL-10-mediated STAT3 activation and increased apoptosis (234–236). SOCS3 hypermethylation was also common in idiopathic myelofibrosis, but not other MPDs, although no significant correlation with survival or other clinical parameters was found, whereas SOCS3 expression was increased in JAK2 V617F-positive myeloproliferative disorders (MPDs) (234–236). SOCS3 was consistently down-regulated in CML cell lines and bone marrow nuclear cells (BMNCs) from CML patients, with enforced expression inhibiting growth (237). Moreover, in cases where SOCS3 was expressed, it enhanced cell survival as a result of BCR-ABL-mediated phosphorylation (238). However, constitutive SOCS3 expression in CML conferred resistance to IFNα (239, 240). Similarly, in CTCL constitutive SOCS3 expression was found to play a protective role by blocking IFN-α mediated growth suppression and differentiation without impacting STAT3 activation (241). Indeed overexpression of SOCS3 in the peripheral blood of non-Hodgkin lymphoma (NHL) patients correlated with advanced disease and a poor response to treatment (242). In one form of this disease, *de novo* follicular lymphoma with t_(14;18)_, SOCS3 expression induced by overexpressed BCL2 was associated with poor prognosis (243, 244). Ablation of SOCS3 within the myeloid lineage was found to promote tumor development in mice. This was mediated by tumor-derived G-CSF driven proliferation of myeloid-derived suppressor cells in the tumor microenvironment *via* STAT3, which suppressed CD8+ T cell responses against the tumor (245). In mice models of colitis associated cancer, SOCS3 deletion in intestinal epithelial cells (IEC) exacerbated the development of cancer through constitutive STAT3 activation leading to IEC proliferation (246, 247). Furthermore, downregulation of SOCS3 was also observed in patients where colitis resulted in carcinogenesis, suggesting that loss of inhibition on STAT3 promotes the development of cancer during colitis (248).

### CISH

CISH is particularly relevant for cytokines that utilize STAT5, being induced largely by STAT5 and predominantly regulating STAT5 activation (19). CISH has been suggested to play a variety of roles outside the immune system, but more recent studies have identified important roles in the regulation of immune cell development and function (30, 249). Intriguingly it appears to serve as a break on both pathological inflammations, as seen in allergy and arthritis, but also anti-tumor responses (49–51).

CISH participates in T cell development mediated through its negative regulation of, principally IL-2 signaling through STAT5 and IL-4/IL-13 signaling through STAT6. This was evidenced from CISH transgenic mice, which showed increased Th2 polarization proposed to be due to suppressive effects of CISH on IL-2 signaling, including STAT5 activation (250), with CISH able to bind to the IL-2 receptor β chain component, which blocks activation of the associated JAK3 (48). A similar mechanism is likely to explain the heightened embryonic lymphopoiesis observed following knockdown of the zebrafish CISH paralogue Cish.a, which also correlated with enhanced STAT5 activation (251). CISH^−/−^ mice suffered from airway inflammation and progressively developed a pulmonary disease characterized by enhanced eosinophils within the airways and epithelial cell hyperplasia (51). This was due, at least in part, to preferential differentiation of T cells into Th2 and Th9 subsets through enhanced IL-4-mediated STAT6 activation, resulting in excessive production of IL-13 and IL-9 by Th2 and Th9 cells, respectively (51) (Figure 4). CISH suppressed activation of STAT6 and STAT5 in response to IL-4 and IL-2 respectively, both of which drive Th9 and Th2 differentiation (51). IL-9 induced CISH expression in CD4+ T cells but CISH did not significantly effect IL-9 signaling, suggesting that IL-9 stimulates CISH in CD4+ T cells for crosstalk inhibition of other cytokines such as IL-4 (252). This contradicted previous studies where CISH overexpression resulted in elevated Th2 expression, possibly due to differences in methodology (250). Alternatively, this could also be due to compensation by other SOCS proteins in response to CISH ablation, causing an imbalance in Th1/Th2 polarization. Indeed human Th2 cells have been shown to express markedly more CISH compared to Th1 cells, supporting a negative regulatory role (253). Lastly, CISH was also able to inhibit IFN-γ signaling to some degree, although this is overshadowed by the much stronger effects of SOCS1 in most lineages, especially CD4+ T cells (254).

CISH also plays important roles in regulating the development of both CD4+ and CD8+ T cells through regulation of T-cell receptor (TCR) signaling, although there is conflicting data regarding the mechanism of action. Enforced expression of CISH in CD4+ T cells markedly increased their proliferation, survival, and activation. This resulted in enhanced MAP kinase activity in response to TCR stimulation, suggesting CISH acts as a positive regulator of TCR signaling, with CISH found to associate with protein kinase C epsilon as a potential mechanism (255). However, deletion of CISH in CD8+ T cells increased their proliferation and activity, including their cytotoxic anti-tumor capabilities (256). This suggested that CISH instead acts as a negative regulator of TCR signaling in these cells, proposed to be mediated by the ability of CISH to degrade the TCR intermediate protein PLC-γ1 rather than by impacting on STAT5 signaling (256, 257).

CISH serves a major role in controlling NK cell production and function, which is mediated by its negative regulation of IL-15. CISH is induced by IL-15, which strongly activates STAT5, and then targets IL-15R-associated JAK1 to mediate degradation of the receptor complex. In the absence of CISH the maturation and cellular turnover of NK cells in response to IL-15 is enhanced (258). However, additional mechanisms are present in order to prevent accumulation of NK cells in the absence of CISH (258). Despite this, CISH deficient NK cells showed enhanced survival and expansion even with low concentrations of IL-2 and IL-15 (259). The hypersensitivity of CISH^−/−^ mice to IL-15 also enhances the anti-tumor effects of NK cells, suggesting that CISH plays a more significant role in the effector functions of NK cells rather than just their differentiation (49). Indeed, in the absence of CISH, IL-15 stimulated increased cytotoxicity of NK cells through the secretion of IFN-γ (49, 259). CISH^−/−^ NK cells additionally displayed enhanced metabolic fitness attributed to increased mTOR signaling complementing their anti-tumor activities, highlighting the multiple roles played by CISH in NK cells (259).

CISH can also influence both DC and macrophage development, in this case through control of GM-CSF and IL-3 mediated STAT5 activity (Figure 5). CISH expression is strongly upregulated during GM-CSF-induced *ex vivo* maturation of bone marrow-derived DC which correlated with high levels of STAT5 activation. Knockdown of CISH in these cells resulted in increased STAT5 activation and enhanced production of DC precursors but impaired maturation of type 1 DCs, which showed reduced ability to stimulate cytotoxic T cells (249). Similarly, in the absence of CISH, GM-CSF stimulated pathological myeloid cell driven inflammation through enhanced responses to inflammatory cytokines while IL-3 stimulated increased myeloid progenitors (50).

CISH has also been implicated in the control of eosinophil function through regulation of IL-13 and IL-5 signaling. CISH was induced by IL-13 in lung fibroblasts *via* STAT6, with CISH ablation leading to increased expression of chemokines such as CCL26 in response to IL-13 to enhance eosinophil migration to the airways (260). Moreover, airway eosinophils expressed CISH at a higher level compared to peripheral blood eosinophils, which correlated with their reduced sensitivity to IL-5 signaling *via* STAT5 (261), with bone marrow cells from CISH^−/−^ mice producing more eosinophils in response to IL-5 (50). It is likely that these effects on eosinophils contributes to the progressive pulmonary disease observed in CISH^−/−^ mice.

CISH has been shown to impact on several models of disease. CISH^−/−^ mice succumbed to experimental allergic asthma at a higher rate than their wildtype counterparts due to enhanced airway inflammation (51). Inflammation was also increased during inflammatory arthritis as well during EAE in CISH^−/−^ mice (50), which also showed resistance to metastasis of tumor cells both *in vivo* and *in vitro* (49). Finally, a variety of CISH single nucleotide polymorphisms (SNPs) have been associated with increased susceptibility to and/or morbidity from a variety of infectious agents. In particular, CISH SNP-292 (rs414171) has been frequently associated with an increase in susceptibility to pathogens such as hepatitis B virus, malaria and tuberculosis infections (262–264). This SNP reduces the expression of CISH in response to IL-2 and is presumed to result in enhanced IL-2 signaling (264). However, how this results in reduced immunity to such a broad range of pathogens remains to be determined.

### Other SOCS Proteins

SOCS4–7 have more pronounced roles outside cytokine signaling (7), particularly in the regulation of receptor tyrosine kinases that mediated the effects of hormones such as insulin and growth factors like epidermal growth factor (EGF) (7).

A specific role for SOCS4 in immune cell development has not been identified, with SOCS4^−/−^ mice showing no significant alterations in steady-state immune cell populations (265). However, these mice were hypersusceptible to influenza virus infection, with delayed viral clearance, impaired trafficking of influenza virus-specific CD8+ T cells and a pathological increase in pro-inflammatory cytokine such as IL-1β and TNF-α, which resulted in early lethality compared to wild-type counterparts (265). The viral susceptibility was shown to be mediated by cells derived from the hematopoietic compartment (265). However, the SOCS4^−/−^ mice showed no difference in CD8+ memory T cell generation or their efficient recall during subsequent infection (266). Furthermore, a recombinant herpes simplex virus (HSV) expressing SOCS4 caused reduced morbidity and mortality compared to standard HSV, which was associated with reduced levels of pro-inflammatory cytokines released mostly by macrophages during the initial innate immune response (267). This suggests that SOCS4 negatively regulates the innate immune response to viral infection to limit the resultant inflammation, although the mechanism of action remains unclear.

SOCS5 has been shown to be constitutively expressed in both B and T lymphocytes and demonstrated to regulate IL-4 signaling *in vitro* (268). However, SOCS5^−/−^ mice do not display any significant abnormalities in their lymphoid compartments including their CD4+/CD8+ ratio (269). Despite this, SOCS5^−/−^ mice showed heightened susceptibility to influenza virus infection, with increased viral titres and heightened pro-inflammatory cytokines particularly IL-6 and G-CSF, resulting in increased neutrophils and enhanced weight loss (270). This was mediated predominantly through non-hematopoietic tissue, with SOCS5 expression in airway epithelial cells increased following influenza infection. SOCS5^−/−^ mice showed hallmarks of enhanced EGF signaling, with SOCS5 shown to be able to target both EGF receptor and the downstream PI3-K subunits, leading to enhanced Akt and STAT3 activation (270). SOCS5 levels have been found be decreased in patients suffering from chronic obstructive pulmonary disorder (COPD) (270), associated with enhanced levels of pro-inflammatory signaling through cytokines such as IL-1β and TNF-α (271). Finally, in a mouse model of allergic conjunctivitis constitutive expression of SOCS5 reduced eosinophil infiltration, possibly due to enhanced IL-4 signaling (268).

There is no evidence of SOCS6 being involved in immune cell regulation, with SOCS6^−/−^ mice not showing any significant abnormalities within their immune system (272). However, there is some evidence that SOCS7 might contribute to immune cell control, since SOCS7^−/−^ mice displayed different immune-related phenotypes dependent on their background. On a C57BL/6 background, SOCS7^−/−^ mice showed a minor increase in neutrophils with around half succumbing to hydrocephalus of unknown etiology (273). In contrast, around half of SOCS7^−/−^ mice on a 129/Sv background developed a severe cutaneous disease characterized by increased mast cell activation, upregulation of IgE and IgG, and mast cells showing increased sensitivity to IgE-induced pro-inflammatory cytokines (274). The signaling pathways involved remained to be identified.

## Therapeutic Applications

Since SOCS proteins play significant roles in the coordination of the immune system, with functions identified in inflammation and immune-related malignancies, their modulation has clear potential for therapeutic intervention. As such, SOCS proteins in various conformations have been developed with some showing promise in pure clinical models. For example a mimetic peptide of the SOCS1 KIR, Tkip has produced efficacious results in the murine EAE model, with inflammatory phenotypes reduced upon administration (275). Similar to the naturally occurring SOCS1, Tkip was found to inhibit IFN-γ signaling and thereby suppress the effector functions of T-cells, ultimately compensating for the low levels of SOCS1 and SOCS3 associated with EAE in mice (275). A different mimetic peptide of SOCS1, R9-SOCS1-KIR was also able to suppress the development of experimental autoimmune uveitis (EAU) in mice with topical administration suppressing the inflammatory activities of IFN-γ, TNF-α, and IL-17, thereby protecting the mice from ocular pathologies (276). Other SOCS1 mimetic peptides have also shown anti-inflammatory effects in mouse models of chronic intraocular inflammatory disease by suppressing the effects of pro-inflammatory cytokines toward retinal cells (277). A cell penetrating SOCS1 (CP-SOCS1) has been found to suppress the induction of pro-inflammatory cytokines by blocking IFN-γ signaling (278). Similar to the endogenous SOCS1, CP-SOCS1 inhibited IFN-γ mediated STAT1 phosphorylation by interacting with IFN-γ pathway components such as JAK2, with the extent of inhibition being dose dependent (278) A cell penetrating SOCS3 (CP-SOCS3) was also developed in order to treat acute liver injury in mice caused by excessive signaling through TNF-α and IFN-γ and found to suppress inflammation driven by inducers such as LPS, limiting the necrosis and apoptosis in the liver (279). CP-SOCS3 was also found to act in a similar manner to its endogenous proteins *in vitro*, although it retained its inhibitory effects for a much longer period of time compared to endogenous SOCS3 (280). Furthermore, deletion of the SOCS box domain in both CP-SOCS1 and CP-SOCS3 further extended their activities and half-life, providing a much more robust anti-inflammatory affect compared to their endogenous counterparts (278, 280).

SOCS proteins have also been shown to impact on immune responses relevant to inflammatory diseases and cancer, which has seen applications to immunotherapy approaches. For example, adoptive transfer of SOCS3-deficient DCs reduced the severity EAE (200), while in mice models of asthma this only slightly alleviated the development of immunogenic tolerance (281). Alternatively, adoptive transfer of SOCS3 deficient macrophages was found to exacerbate the development of acute lung injury in mice, emphasizing the multifaceted roles of SOCS3 (220). Lastly, modulation of CISH was found to be effective in protecting against tumor subtypes under the control of NK cells (282). SOCS1 ablation in human DCs was shown to be required to break self-tolerance in order to induce anti-tumor responses (283), resulting in enhanced activation of DCs, increased IFN-γ and enhanced killing by cytotoxic T cells (284, 285). In addition, treatment with tumor cell-conditioned media was shown to upregulate SOCS1 to suppress DC maturation (286). Furthermore, SOCS1 silencing in macrophages also suppressed tumor development due to enhanced anti-tumor inflammation (287). In contrast, adenovirus delivery of SOCS1 was shown to enhance T cell-mediated anti-tumor immunity (288). Combination therapies involving CISH inhibition, and BRAF as well as MEK inhibitors can extend the anti-tumor effects to additional subtypes, providing additionally opportunities for CISH modulation (282).

## Conclusion

It is evident that SOCS proteins represent an important intrinsic mechanism for maintaining homeostasis especially within the immune system. As such it is unavoidable that their dysregulation significantly affects the delicate and complex processes that govern immunity leading to a variety of diseases particular inflammation, autoimmunity and cancer. Hence, it is vital that the various pathways regulated by SOCS proteins be identified to better understand the conditions caused due to their dysregulation. While linear pathways involving one cytokine and SOCS protein are now well-understood, the complex interplay between multiple pathways which ultimately controls cellular responses awaits considerable further attention. Moreover, it is important to look beyond the initial notions that the influence of SOCS proteins is merely inhibitory as recent evidence suggests that SOCS proteins can also have a stimulatory effect on some pathways. Moreover, the vast majority of studies regarding SOCS proteins have focused on CISH and SOCS1–3, while SOCS4–7 have only been studied in a handful of papers. While CISH and SOCS1–3 are clearly more important with regard to the immune system, recent studies have shown that SOCS4–7 likely also contribute, warranting additional studies on these proteins. Lastly, the targeting of SOCS proteins for therapeutic purposes continues to show promise. Therefore, there remains much work to do to fully understand the SOCS proteins and harness them to fight disease.

## Author Contributions

MS and AW contributed to the conception of the review. MS wrote the first draft of the manuscript and prepared the initial figures with assistance from CL. AW contributed additional material for the manuscript. All authors contributed to manuscript revision, read, and approved the submitted version.

## Conflict of Interest

The authors declare that the research was conducted in the absence of any commercial or financial relationships that could be construed as a potential conflict of interest.

## Publisher's Note

All claims expressed in this article are solely those of the authors and do not necessarily represent those of their affiliated organizations, or those of the publisher, the editors and the reviewers. Any product that may be evaluated in this article, or claim that may be made by its manufacturer, is not guaranteed or endorsed by the publisher.
